# Comparing Staging Systems for Predicting Prognosis and Survival in Patients with Hepatocellular Carcinoma in Egypt

**DOI:** 10.1371/journal.pone.0090929

**Published:** 2014-03-06

**Authors:** Asmaa Ibrahim Gomaa, Mohamed Saad Hashim, Imam Waked

**Affiliations:** Hepatology Department, National Liver Institute, Menoufiya University, Shebeen Elkom, Menoufiya, Egypt; University of Pisa, Italy

## Abstract

**Introduction:**

Several hepatocellular carcinoma (HCC) staging systems are available. Although the European Association for Study of Liver Diseases (EASL) and American Association for the Study of Liver Diseases (AASLD) recommended the use of Barcelona Clinic Liver Cancer (BCLC), many studies in different populations revealed heterogeneous results. The aim of this study was to compare different staging systems for predicting prognosis and survival, and for stratifying HCC patients for treatment at a national referral centre for liver disease in Egypt.

**Methods:**

2000 Patients were included in this study. Baseline demographic, clinical, laboratory, and radiological data were determined at diagnosis. Patients were stratified using the Okuda, BCLC, Cancer of the Liver Italian Program (CLIP), and Japan Integrated Staging (JIS). Patients’ survival in different stages within each staging system and the validity of the system in predicting survival were compared.

**Results:**

The overall survival was 15 months. The 1-, 2-, 3- and 4-year survival of the entire cohort was 56%, 34%, 25% and 15% respectively. The presence of ascites, multiple focal lesions, large tumour size >5 cm, portal vein thrombosis, extra-hepatic spread, AFP≥200 ng/ml and poor Child score were independent predictors of survival (*p*<0.001). All staging systems were significant in determining overall survival in univariate and multivariate analyses. BCLC was the most predictive staging system for the whole cohort (*p*<0.001). Among the subgroup of patients offered potentially curative therapy, BCLC was the most informative system in predicting patient survival (*p*<0.001). For patients with advanced HCC not amenable for specific therapy, CLIP was the best staging system for predicting prognosis (*p*<0.001).

**Conclusion:**

BCLC staging system provided the best prognostic stratification for HCC patients. However, CLIP score has the highest stratification ability in patients with advanced HCC highlighting the importance of including AFP in best staging system.

## Introduction

Hepatocellular carcinoma (HCC) is the commonest primary malignant liver tumor The incidence of HCC is increasing all over the world, and it causes about 690.000 mortalities every year, ranking third in the cause of cancer deaths [1], [2].

In Egypt, hepatocellular carcinoma is the second most common malignancy in males and the fifth in females [3]. There was almost a twofold increase of the proportion of HCC among chronic liver disease patients in Egypt in the past ten years with a significant decline of HBV and slight increase of HCV as risk factors [4].

HCC is unique in comparison with other cancers in that the presence of chronic liver disease and cirrhosis affects the ability to treat the tumor and the overall patient survival. Therefore, liver disease is a very important variable, together with the overall health of the patient [5], [6].

Tumor staging at the time of diagnosis is essential to determine the patients overall survival probability prior to treatment, decide which type of therapy is the most appropriate and enable objective comparison among the outcomes of research studies [6].

Several staging systems for HCC have been proposed as the Okuda [7], TNM [8], the Cancer of the Liver Italian Program (CLIP) [9], the Groupe d’Etude et de Traitement du Carcinome Hepatocellulaire Prognostic classification (GETCH) [10], Barcelona Clinic Liver Cancer (BCLC) [11], Vienna classification [12], Chinese University Prognostic Index (CUPI) [13], Japan Integrated Staging (JIS) score [14] and Tokyo [15] staging system.

These staging systems used variables that can be grouped into four aspects: tumour factors, factors related to underlying liver function, overall health of the patient and efficacy of treatment. However, with >15 HCC staging classifications available, each measuring a range of different factors and developed in different patient populations, physicians are confused which classification to use.

There has been an intense debate over the past decade for choosing an optimal staging system for HCC owing to the wide variation in patient selection and preferred treatment modality in different published studies. The most reliable and widely adopted methods for staging HCC are the CLIP and BCLC systems in Europe and the JIS in Japan. They have been internally and externally validated, both retrospectively and prospectively, and their efficiency has been evaluated in several clinical and therapeutic studies [16]–[24]. The BCLC is endorsed by the EASL and the AASLD. Similarly, the biomarker-combined JIS score is the standard Asia-Pacific classification system for HCC.

The aims of this study were to identify the independent predictors of survival at the time of HCC diagnosis and to compare the accuracy of commonly used HCC staging systems in predicting survival in a cohort of Egyptian patients with HCC to select the best staging system for Egyptian patients then to evaluate the performance of this system in different subgroups of patients according to offered treatment.

## Patients and Methods

### Ethics Statement

This observational study was approved by the institutional review board of the National Liver Institute (IRB number IRB00003413). The participants provided written informed consent to participate in this study. The IRB approved this consent procedure.

The study was conducted prospectively in the period from January 2010 to December 2012 on 2000 patients diagnosed with HCC in the National liver Institute, Menoufiya University, Egypt. The diagnosis of HCC was based on histological study of tumor tissue taken from resected or biopsied samples in 15 patients and on non-histological criteria in other patients according to AASLD guidelines [25].

For all patients, demographic information, etiology of liver disease, biochemical data including serum bilirubin, serum albumin, prothrombin time and concentration, ALT, AST, complete blood and serum alpha-fetoprotein (AFP) were evaluated. Presence of underlying cirrhosis, ascites and encephalopathy were assessed. Assessment of hepatic function based on Child-Turcotte-Pugh (CTP) was recorded. The number and location of nodules, maximum diameter of the largest nodule, presence of portal vein thrombosis and extrahepatic metastasis were recorded.

For most patients who were categorized at presentation in an advanced stage, this was due to presence of vascular invasion and portal vein thrombosis or abdominal lymph node spread on initial abdominal CT. During initial assessment, a chest X ray was done, and if abnormal, a CT chest was done. Bone-scan or CT brain was done if there was any suggesting symptoms or clinical indication.

Staging of the tumor was determined at the time of HCC diagnosis using the Okuda, CLIP, BCLC and JIS staging systems. All patients were monitored from the time of diagnosis to the date of death or date of data collection if they remained alive.

### Statistical Analysis

Overall survival of patients was the single end point used to assess the performance of the different staging systems. Length of survival was calculated from the date of HCC diagnosis to the date of death or, in the case of survivors, the date of the last follow up visit. Continuous data were expressed as the mean ± SD. A univariate analysis to identify predictors of survival at the time of HCC diagnosis was performed using the Kaplan-Meier method of survival function [26].

Survival curves were estimated by the Kaplan-Meier method and compared, for univariate analysis, by the log rank test. For evaluation of continuous variables, patients were divided in two groups based on the median value in the study group as a whole. Parameters that proved to be significant in univariate analysis were tested subsequently with the multivariate Cox proportional hazard model [27] to identify independent predictors of survival.

The performance of a prognostic system [28] has been shown to be related to homogeneity (small differences in survival among patients in the same stage within each system), discriminatory ability (greater differences in survival among patients in different stages within each system), and monotonicity of gradients (the survival of patients in earlier stages is longer than the survival of patients in more advanced stages within the same system).

The prognostic performance of each scoring system was statistically assessed, evaluating homogeneity within classification groups, discriminatory ability, and monotonicity of the gradients in the association between stages and survival rates. Thus we used a multistep approach. Firstly we evaluated, at univariate analysis, the capacity of each score to distinguish categories of patient with significantly different survival (homogeneity of the staging system). For each system, this performance was evaluated by comparing by log rank test the survival curves of the single categories, calculated using the Kaplan-Meier method. Next, we needed to compare the overall predictive power of survival for each staging system to assess which gave the most accurate prediction of survival (monotonicity of the system). This point was evaluated by the linear trend χ2 test, entering each scoring system into a Cox regression model.

Finally, to evaluate the discriminatory ability for the prediction of survival, we evaluated the accuracy of prediction of death at one, two and three years for each scoring system. This point was evaluated calculating the area under the receiver operating characteristic (ROC) curve for each staging system (which is equivalent to the concordance statistic (c statistic)) [29]. To perform this test, patients censored before one, two, and three years were excluded from the analysis. P values <0.05 were considered statistically significant and P<0.0001 was considered to be of high statistical significance. Statistical analysis was performed using SPSS 17 for Windows.

## Results

The patients constituted 1678 (84%) males and 322 (16%) females, with an age range of 20–84 years (median age, 56 years). Table 1 shows the demographic, clinical, and tumor information for all patients.

**Table 1:** Characteristics of all patients included in the study.

Variable		Value
Age, years	Median	56
	Range	20–84
	Mean ± SD	56±8.8
Male, n (%)		1678 (84)
Presentation, n (%)	Follow up of liver cirrhosis	660 (33)
	Complication of liver cirrhosis	780 (39)
	Accidental discovery	560 (28)
Mode of diagnosis, n (%)	Histology	15 (0.8)
	Imaging+tumor marker	1985 (99.2)
Cirrhosis, n (%)		1802 (90)
Ascites, n (%)		799 (39.9)
Etiology of liver disease	HCV	1740 (87)
	HBV	40 (2)
	Cryptogenic	100 (5)
	Missed data	120 (6)
Number of tumor nodule, n (%)	One	959 (47.9)
	Two	291 (14.6)
	Three	303 (15.1)
	Multiple	447 (22.4)
Size of nodule,	M ± SD	6.9±3.9
	Range, cm	1–21
Location of nodule, n (%)	Right	1165 (58.3)
	Left	366 (18.3)
	Both lobes	469 (23.4)
Portal vein thrombosis, n (%)		379 (19)
Metastasis at diagnosis, n (%)		111 (5.5)

Most patients had liver cirrhosis (90%); mainly due to chronic hepatitis C infection; and 40% had ascites. The number of tumors was determined from the pretreatment triphasic CT scan. Nine hundred and fifty nine patients had a single focal lesion, and 23.4% had both right and left lobes lesions. Tumor size was determined as the greatest dimension of the tumor measured on the pretreatment CT scan. The size of the focal lesion ranged from 1 cm to 21 cm and the tumor diameter was greater than 5 cm in 56% of patients. Vascular invasion was assessed by ultrasound, dynamic CT or hepatic angiography. There was portal vein thrombosis in 19% of patients at the time of diagnosed. Lymph node invasion or distant metastases was detected in 111 (5.5%) patients at diagnosis.

Table 2 shows the stages of the disease by using different staging systems and scores, and the treatments offered. Surgery or radiofrequency ablation (RFA) were reserved for patients with single lesions less than 5 cm, or with up to three lesions, each less than 3 cm. Transarterial chemoembolization (TACE) was applied to patients with single lesions larger than 5 cm or with multiple lesions involving less than 50% of the liver parenchyma or to patients with a single lesion less than 5 cm which was difficult for local ablation under ultrasound guidance (superficial subcapsular lesion). Supportive care alone was provided to patients with multifocal bilobar disease and/or vascular invasion and Child C patients. Sorafenib was recommended to patients with child A with vascular invasion or distant metastasis and good performance status. Only few patients received a liver transplant, as liver transplants are only performed from living donors in Egypt. In our center, patients are referred for transplantation if the HCC is within the Milan criteria, and the AFP is below 1000. Patients below the age of 60 and with a willing related donor were offered transplantation, and those who accepted were referred to the transplant unit for evaluation, where the donor acceptance rate is low (around 15%).

**Table 2:** Staging by different systems and scores, and treatments offered.

Variable		Value
Child-pugh, n (%)	A	714 (35.7)
	B	800 (40)
	C	486 (24.3)
Okuda, n (%)	I	696 (34.8)
	II	917 (45.8)
	III	387 (19.4)
CLIP, n (%)	0	271 (13.6)
	1	507 (25.3)
	2	531 (26.5)
	3	324 (16.2)
	4	212 (10.6)
	5	123 (6.2)
	6	32 (1.6)
BCLC, n (%)	A	501 (25)
	B	608 (30.4)
	C	405 (20.3)
	D	486 (24.3)
JIS, n (%)	0	27 (1.3)
	1	361 (18)
	2	625 (31.2)
	3	579 (29)
	4	331 (16.6)
	5	77 (3.9)
Treatment modality, n (%)	Resection	117 (5.9)
	Transplantation	38 (1.9)
	PEI	72 (3.5)
	RFA	283 (14.1)
	TACE	749 (37.5)
	Microwave ablation	22 (1.1)
	Sorafenib	176 (8.9)
	Supportive	543 (27.1)

### Overall Survival

At the time the data were censored, 962 (48%) patients had died. The overall median survival of the entire cohort for a 3 year follow-up period was 15 months (95% CI: 13.6–16.3 months) and the 1-, 2- and 3- year probability of survival was 55%, 33%, and 25% respectively.

### Univariate Analysis

Overall survival was compared for 12 possible prognostic factors, including 7 baseline patient factors (age, sex, presence of ascites, albumin level, total bilirubin level, prothrombin time, and Child-Pugh class) and 5 baseline tumor factors (serum alfa-feto-protein level, multiplicity of tumors, greatest tumor dimension ≤ or >5 cm, portal vein tumor thrombosis and extrahepatic metastasis).

Using the Kaplan-Meier method, univariate analysis showed that presence of ascites, presence of two or more neoplastic nodules, maximum tumor diameter >5 cm, portal vein thrombosis, extrahepatic metastasis, AFP≥200 ng/ml, CTP class B or C were significantly associated with poor survival in patients with HCC (Table 3).

**Table 3:** Univariate analysis of baseline predictors of survival.

Variable		Median survival (months)	*P* value
Age	<56	15	0.24
	≥56	16	
Sex	Male	15	0.09
	Female	17	
Ascites	Absent	20	<0.001
	Present	8	
Number of tumor nodule	One	20	<0.001
	Two	16	
	Three	11	
	Multiple	8	
Maximum tumor diameter	≤5 cm	26	<0.001
	>5 cm	12	
PVT	Absent	19	<0.001
	Present	6	
Extrahepatic metastasis	Absent	16	<0.001
	Present	6	
AFP level	<200	20	<0.001
	≥200	11	
Child-Pugh score	A	30	<0.001
	B	15	
	C	5	
Treatment modality	Resection	36	<0.001
	Transplantation	12	
	PEI	20	
	RFA	Undefined	
	TACE	18	
	Microwave ablation	Undefined	
	Sorafenib	7	
	Supportive	6	

*P* value <0.05 is significant.

Patients who received treatment for HCC had significantly better survival compared with those who did not receive treatment (*p*<0.001). Treated patients had a median survival of 20 months in contrast to 6 months in those untreated.

### Multivariate Analysis

The seven factors which were significant in the univariate analysis were entered in multivariate analysis (Cox proportional hazard regression) as shown in Table 4. The presence of ascites, multiple focal lesions, large tumour size, portal vein thrombosis, extrahepatic spread, AFP level and CTP score were independent predictors of survival.

**Table 4:** Multivariate analysis of baseline predictors of survival.

Variable	Hazard ratio	95% CI	*P* value
Number of tumor nodule>one	1.17	1.01–1.35	0.02
Maximum tumor diameter ≥5 cm	1.78	1.53–2.07	<0.001
PVT	2.08	1.76–2.45	<0.001
Extrahepatic metastasis	1.65	1.28–2.12	<0.001
Poor Child score	2.31	1.93–2.75	<0.001
AFP≥200	1.32	1.14–1.51	<0.001
Presence of ascites	1.61	1.39–1.88	<0.001

*P* value <0.05 is significant.

### Performance of the Staging Systems

Table 5 shows the results of staging of all studied patients using the Okuda, CLIP, BCLC and JIS systems, with median survival times and survival probability at one, two and three years respectively.

**Table 5:** Patient survival according to different staging systems.

Staging system	Stage	1-year survival (%)	2-year survival (%)	3-year survival (%)	Median survival (months)	*P* value
Okuda	I	77%	58%	47%	30	<0.001
	II	52%	23%	14%	13	
	III	23%	9%	7%	6	
CLIP	0	86%	69%	58%	32	<0.001
	1	76%	53%	39%	27	
	2	57%	25%	15%	15	
	3	38%	7%	6%	9	
	4	22%	10%	5%	7	
	5	9%	0%	0%	5	
	6	0%	0%	0%	3	
BCLC	A	90%	69%	56%	32	<0.001
	B	69%	33%	18%	18	
	C	26%	5%	4%	7	
	D	17%	6%	0%	5	
JIS	0	95%	84%	75%	31	<0.001
	1	87%	69%	57%	31	
	2	71%	39%	27%	20	
	3	45%	16%	4%	12	
	4	14%	9%	9%	6	
	5	9%	2%	0%	4	

*P* value <0.05 is significant.

**Figure 1:**
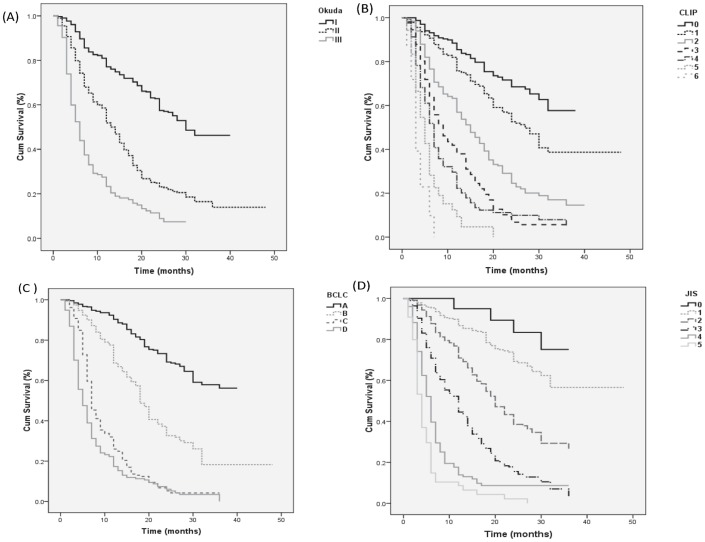
Survival curves for whole cohort. Kaplan-Meier survival curves for patients with hepatocellular carcinoma by staging system: (A) Okuda, (B) Barcelona Clinic Liver Cancer, (C) Cancer of the Liver Italian Program, (D) Japan Integrated Staging score.

The staging systems were analyzed separately using Kaplan–Meier survival analysis. Each staging system showed a significant difference in the probability of survival across the different stages (Fig. 1). All staging systems showed significantly improved survival in patients with early stage disease compared to patients with advanced stage disease.

The individual pairwise comparisons revealed that the Okuda, CLIP and BCLC systems had a better stratification of survival across all stages (*P*<0.01). The JIS score had a better stratification of survival across stages 1, 2, 3, 4 and 5 (*P*<0.01) although it had poor stratification of survival at the early stages (stages 0 and 1) (*p* = 0.14).

When entered into a Cox regression model, the BCLC staging system showed better performance in prediction of overall survival compared with the CLIP, JIS and Okuda (Table 6). The BCLC system had the highest homogeneity (LR χ^2^ 810), indicating small differences in survival among patients in the same stages.

**Table 6:** Likelihood ratio on entry of individual staging systems as factors in Cox regression model for the whole cohort.

Staging system	LR χ2 test	−2 log likelihood	*P* value
Okuda	363	12869	<0.001
CLIP	679	12663	<0.001
BCLC	810	12473	<0.001
JIS	694	12620	<0.001

LR, likelihood ratio, *P* value <0.05 is significant.

**Figure 2:**
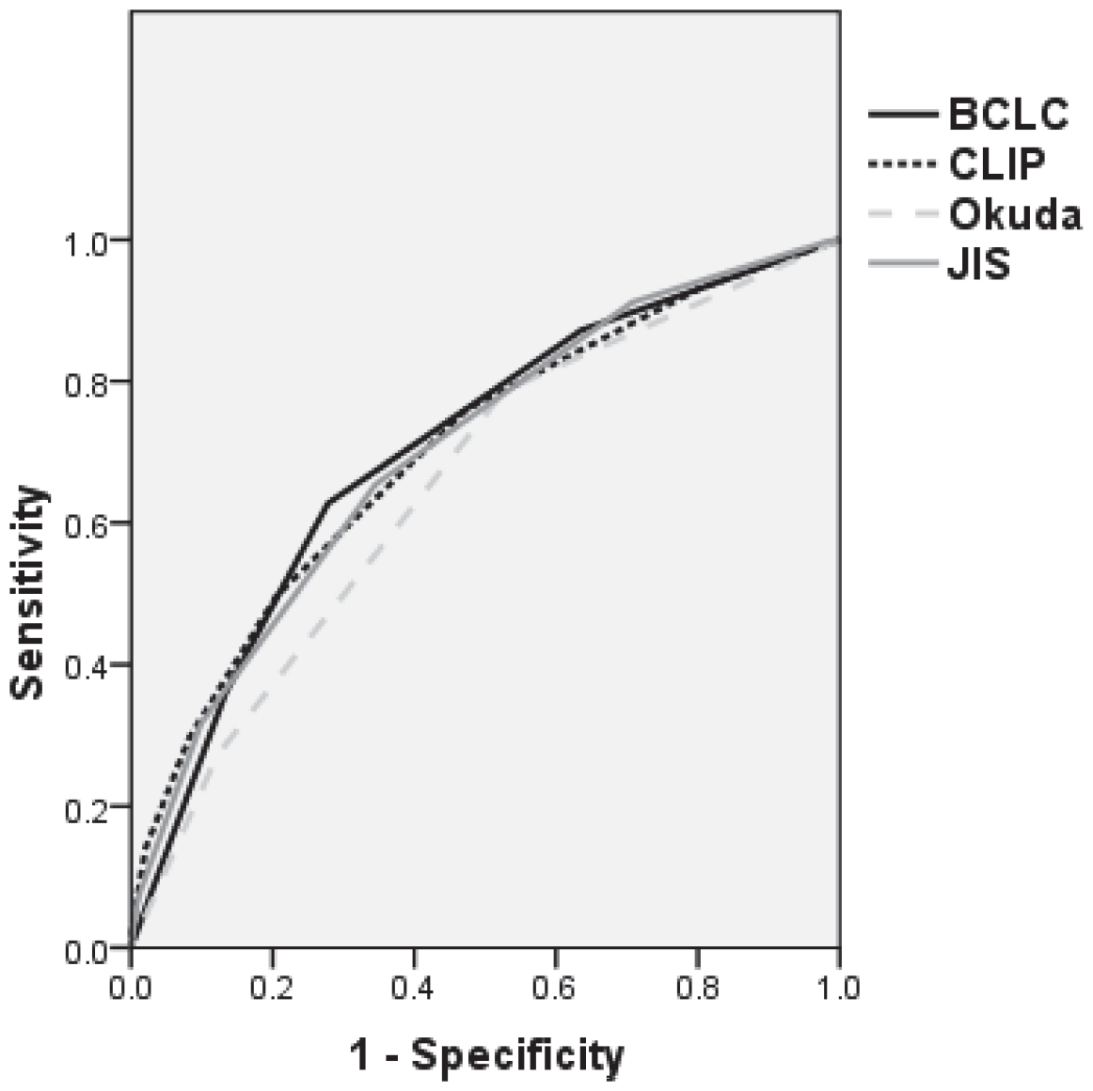
Receiver operating characteristic (ROC) curve for whole cohort. Discriminatory ability for death at one, two, and three years, evaluated by receiver operating characteristic (ROC) curve area, for Okuda, CLIP, BCLC and JIS scores for whole cohort.

Discriminatory ability for death of the entire cohort, evaluated by ROC curve area analysis, was higher for BCLC and closely followed by CLIP and JIS compared with Okuda (Fig. 2). The area under curve for BCLC was 0.705 (CI 0.682–0.727).

### Predictors of Survival in Patients not Amenable to Specific Treatment

Seven hundreds and nineteen patients were not amenable to specific treatment; defined as those who were not candidates for surgical or locoregional ablation. More than half (69.3%) died before the end of this study. The median survival time was 7 months and the 3-, 6-, 9- and 12- month survival rates were 80%, 50%, 31% and 22% respectively.

**Figure 3:**
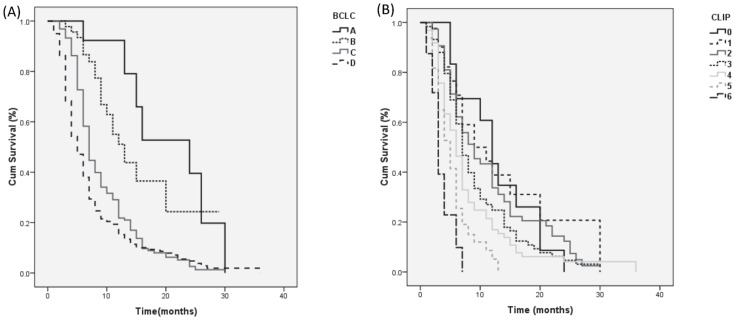
Survival curves for patients not amenable to treatment. Probability of survival for patients not amenable to treatment according to (A) CLIP score, (B) BCLC staging system.

Both the CLIP and BCLC systems identified distinct subgroups with a different prognosis within those patients. BCLC restaging of the patients that were not amenable to treatment revealed a subgroup of 15 patients whose tumors were classified as BCLC stage A and who showed a mean survival longer than that observed in the whole group (24 months vs 7 months). Another subgroup of 48 patients had BCLC stage B and median survival of 13 months. In contrast, the 379 patients classified as BCLC stage D had a worse prognosis than that predicted by the median of the group as a whole: 5 months vs 7 months (Fig. 3A). Using the log rank test, significant differences were found among survival rates of subgroups with stage B, C and D tumors (*p*<0.001), however, no significant difference between stages A and B were noticed.

CLIP restaging of the those patients revealed 19, 53 and 161 patients whose tumors were classified as CLIP stage 0, 1 and 2 respectively and who showed a longer median survival time than that observed in the whole group (12, 9 and 9 months vs 7 months). Also, 160, 112 and 32 patients classified as CLIP stage 4, 5 and 6 respectively had worse prognosis than that predicted for the whole group (6, 5 and 3 months vs 7 months). Kaplan Meier analysis showed significant differences among survival rates of subgroups with stage 3, 4, 5 and 6 tumors (*p*<0.01). However, there no significant differences were found among subgroups with stage 0, 1, 2 and 3 (Fig. 3B).

Cox regression analysis showed that the CLIP score had better performance in prediction of overall survival compared to the BCLC in this subgroup of patients. The CLIP system had the higher homogeneity (LR χ^2^ 111) compared to BCLC (LR χ^2^ 59), indicating small differences in survival among patients in the same stages.

**Figure 4:**
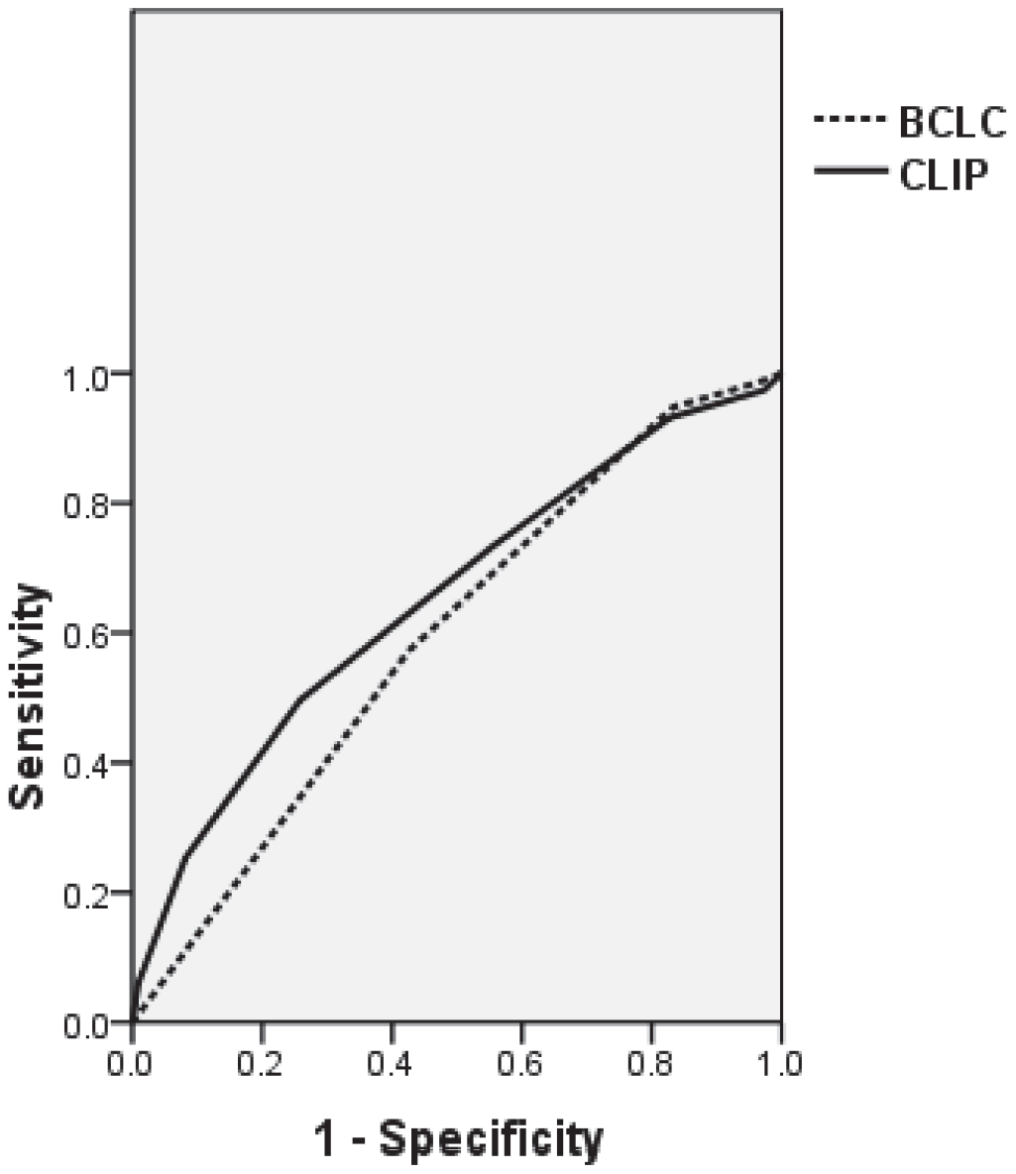
ROC curve for patients not amenable to treatment. Discriminatory ability for death at one, two, and three years, evaluated by receiver operating characteristic (ROC) curve area, for CLIP and BCLC staging system for patients not amenable to treatment.

Using ROC curve analysis, the area under curve was higher for CLIP (AUC = 0.652, CI 0.610–0.694) compared with BCLC (AUC = 0.594, CI 0.548–0.640). CLIP showed a significantly better discriminatory ability (Fig. 4).

### Predictors of Survival in Patients Amenable to Potentially Curative Treatment

Patients who were suitable for resection, liver transplantation or RFA had a median survival time of 36 months and the 1-, 2- and 3- year survival rates of 81%, 64% and 49% respectively. Kaplan Meier analysis showed significant differences among survival rates of BCLC stages A, B and C tumors (P<0.001), however no significant difference found between BCLC stages C and D (p = 0.59). Also, significant differences among survival rates of CLIP subgroups with stage 0, 1, 2 and 3 tumors (*p*<0.01) were found. However, there were no significant differences within stages 3, 4 and 5 (*p* = 0.76 and 0.97).

Multivariate analysis showed that the BCLC had better performance in prediction of overall survival in this group compared to the CLIP score. The BCLC had LR χ^2^ of 132 compared to CLIP score (LR χ^2^ 73). The discriminatory ability of BCLC improved when analysis was done for patients amenable to curative treatment rather than for whole cohort. Using ROC curve analysis, the area under curve was higher for BCLC compared to CLIP (AUC = 0.647, CI 0.593–0.701 vs 0.642, CI 0.589–0.695).

## Discussion

Management of HCC patients has improved owing to updates in diagnosis, patient care and treatment outcomes. This necessitates improving prediction of the prognosis in order to properly identify the potential candidates for therapy.

Despite the high prevalence and mortality of HCC in Egypt, no published study had stratified the survival outcomes, evaluated which of the existing tumor staging systems has the best prognostic value for HCC and its impact on choice of different treatment modalities in a cohort of Egyptian patients.

Design of a tumor staging system depends on the identification of individual prognostic variables that can predict survival of patients with HCC. We collected data and followed a large cohort of patients with HCC (2000 patients) to study prognostic factors for HCC patients in Egypt. In this study 36% of patients had advanced liver disease and received supportive treatment while the rest of patients had early or moderate disease.

Mean duration of survival in the total population was 15 months. Univariate analysis and multivariate analysis showed that the independent predictors of survival were the extent of tumor (tumor size, number of nodules, extrahepatic spread, AFP level and portal vein involvement), hepatic function (absence of ascites and good child score) and the treatment modality.

There was no significant correlation between age and survival in our study. Many reports indicate that female HCC patient more frequently have a well-encapsulated, less invasive tumor, longer survival, lower recurrent rate and better prognosis than male patient, which might be due to the receptor of sex hormones [30]. However, gender did not significantly affect the survival in our study as well in other studies [6], [16], [24], [31].

Tumor burden had been shown to be an independent prognostic factor in previous studies. The cutoff used in previous studies has varied from more than 2 cm diameter of the largest nodule to a tumor involving more than 50% of the liver [6], [24], [32]. We used 5 cm as cutoff, and most of our patients presented with large tumors, with more than 50% having tumors >5 cm in diameter. We found that tumors more than 5 cm diameter correlated significantly with poor survival.

The prognosis of patient with single tumor nodule is much better than those with multiple nodules. In this study, the number of nodules was a significant baseline predictor of survival. Patients with multiple focal lesions had significantly worse survival. This is consistent with previous reports [6], [24], [31], [32].

Portal vein thrombosis had been found to be an important prognostic indicator [6], [24], [32], [33]. Portal vein involvement was associated with worsened hepatic function, high rate of recurrence after ablation and bad prognosis. In our cohort, 19% had portal vein thrombosis at the time of diagnosis, and there was a highly significant negative correlation between PVT and survival.

The correlation between AFP level and the severity of HCC has been investigated in multiple studies [16], [34]–[37]. Serum AFP is useful as a prognostic indicator for HCC patients at the time of tumor diagnosis [37]. Patients with a normal AFP level have a lower incidence of tumoral vascular invasion and tend to present better hepatic function. This may be due to the fact that well-differentiated tumors express less AFP [36]. We found that AFP level ≥200 ng/ml was significantly associated with poor survival.

At the time of diagnosis of HCC, 5% of patients had extrahepatic metastasis in our study. We found a significant negative correlation between the presence of extrahepatic spread at the time of HCC diagnosis and the overall survival. Other studies [16], [24] found the same correlation.

As most of our patients had underlying cirrhosis, the survival was found to be related to hepatic functional reserve. In our study, poor Child score or presence of ascites were significantly correlated with poor survival. This is consistent with previous reports [34], [38]–[42].

In our study, patients treated with resection had a better survival (36 months) than patients treated with tumor ablation (20–27 months) or those treated conservatively (6 months). However, this may be due to the variations in the patients’ characteristics.

Tumor staging at the time of diagnosis is essential to identify cases amenable to treatment and decide the most appropriate therapy [6]. The BCLC system was validated as the most suitable prognostic system for patients with HCC in Italy and the United States [16], [24]. Also, the CLIP staging system is a clinical scoring system that accounts for both liver function and tumor characteristics. The CLIP system has been externally validated in Canadian [43], Italian [23], and Japanese cohorts [28].

Our study represents the first independent study examining HCC prognostic classification systems in a cohort of Egyptian patients referred to the National liver Institute. This study included a cohort of HCC patients enrolled over three years who and prognostic staging were determined for each patient before treatment initiation.

Our analysis of the four tumor staging systems for HCC demonstrated a progressive decrease in survival rates from the earliest to the most advanced stage. However, the BCLC system was the best at discriminating survival of patients in different stages and had the greatest homogeneity of survival among patients within the same stage. We found that each scoring system has a significant correlation between survival and tumor stage (*p*<0.001). Using Cox regression model, the BCLC system had the best performance in prediction of overall survival compared to the Okuda, BCLC and JIS. Discriminatory ability for death evaluated by ROC curve area analysis was higher for BCLC compared to CLIP, JIS and Okuda.

The applicability of staging systems of HCC may be dependent on the offered treatment and the predictive power of a staging system may be altered in patients treated differently. For example, the best staging system for HCC patient who undego surgery might not be suitable for patients who receive only supportive care. In our study, both the BCLC and CLIP systems proved to distinguish subgroups of patients according to offered treatment. The performance of BCLC was better than that for whole cohort when the analysis was applied on a subgroup of patients offered potentially curative therapy. BCLC was the most informative system in predicting survival for these patients. However, when separate analyses were performed for those patients not amenable to curative treatment, CLIP system appeared to be superior to BCLC using univariate and multivariate analysis. CLIP had higher AUC than BCLC, indicating that it provides better stratification of late stage HCC patients. In the era of targeted therapy, proper patient selection is an important issue to help the success of clinical trials of new agents, as a 3-month life expectancy is an essential inclusion criterion. CLIP score offers this requirement.

As any staging model is constructed from selected risk factors of certain stage of HCC in a specific population, the predictive power of this staging model could be considerably impaired if it is applied to another population where a majority of patients do not have the same stage HCC, as the clinical outcome is closely associated with patient demographics and subsequent treatment strategy. Multiple studies comparing staging systems in hepatocellular carcinoma have been conducted, including patients with different stages of HCC, and have reported different ranking of staging systems [6], [17], [24], [44]–[47]. The CLIP and BCLC were the top ranking systems in most studies. The CLIP system was originally derived from a large unselected patient population the majority of whom had been treated conservatively, while BCLC was derived from surgically oriented centres [48]. Therefore, the BCLC system was better than the CLIP and Okuda systems in some studies [6], [17], [48] while CLIP was more informative in some studies [38], [44], [47], [49], [50] and JIS was the best in other studies [51]–[53]. Several factors may contribute to these discrepancies. The characteristics of tumor-related variables, preferred treatment modality in different centers, the number of patients analyzed, the pattern of patient referral and the clinical characteristics and the etiology of cirrhosis could all or in part influence the final results.

Our findings confirm the results of other studies. A prospective study conducted on 195 patients with HCC included a large proportion of patients who had potentially curative therapies [16]. The authors found that BCLC had the best independent predictive power for survival when compared with the Okuda, CLIP, UNOS-TNM and JIS systems. When patients treated by liver transplantation were excluded from the survival analysis, the prognostic performance of CLIP and Okuda scores improved dramatically.

A recent retrospective study conducted to compare six prognostic staging systems (Okuda stage, TNM stage, CLIP score, BCLC stage, JIS score and Tokyo score) in predicting survival in 2010 patients with HCC in a single center in Taiwan over twenty years [54]. The Tokyo score was the most informative one for predicting survival of HCC patients as a whole, receiving surgical resection, or receiving transarterial chemoembolisation. CLIP score was the best system for HCC patients receiving chemotherapy or supportive care.

Another study conducted on 187 patients with advanced HCC in medical oncology unit [49] revealed that CLIP score was more informative than BCLC in predicting survival in those patients owing to the fact that patients with advanced HCC have distinct clinical characteristics, tumor extent, and residual liver function.

Our study is the first single-centre study carried out in Egypt where patient demographics and aetiology of HCC are different from that in Europe and in Asia. Large number of our studied population had advanced disease who were not amenable to curative or local therapies.

In general, the BCLC system contains treatment derived parameters and is effective in areas where HCC is diagnosed at relatively early stages where surveillance programs are applied, whereas the CLIP system is the best for patients with intermediate or late stage disease, suitable for clinical trials. Thus it is necessary to take into consideration the varying demographic characteristics of patients, including ethnicity, all known predictive factors, from early to advanced stages, within the different cohorts for building an ideal staging system to fit all patient populations. Such discrepancies necessitate the need for modification of available staging systems to account for different cohorts.

It is well established that identification of prognostic factors within a population is the corner-stone in proposing staging systems. It was found that tumor burden, Child Pugh score, PVT, AFP level were independent prognostic factors of survival in Egyptian HCC cohort in this study. In addition, AFP is a variable in CLIP score that is not included in BCLC. So adding AFP to variables included in BCLC may improve its performance.

Recently, there were improvements in staging systems with formation of new editions in the different prognostic scores, which appear to have better prognostic stratification than older ones [55]–[57]. However, efforts to construct a universally applicable staging system always fail because this approach would neglect the epidemiologic, etiologic and other geographic parameters of HCC beyond the parameters incorporated in the staging systems. Therefore, it is mandatory to obtain a validated region-oriented staging system [58].

Nowadays, genomic and proteomic studies has revolutionized the understanding of the molecular basis of HCC; therefore, many studies aimed to discover molecular biomarkers for cancer staging for prediction of prognosis and for treatment selection. However, this technology is too expensive to be studied in large number of cases. Therefore, until this new technology becomes an established method in the prediction of the prognosis of patients with HCC, we should depend on clinical staging systems [59].

In conclusion, our results confirm that BCLC is a good prognostic system and that CLIP score is the best for patients not amenable to treatment. Incorporating both BCLC and CLIP staging systems in one system may improve their performance. As all factors in CLIP score are present in BCLC except AFP, the addition of AFP may improve the BCLC system for Egyptian patients.

Limitation of this study is the relatively few patients included in the early stages affecting its value for surgical cohorts.
